# Burnout Syndrome among Staff at an Emergency Department during the COVID-19 Pandemic

**DOI:** 10.3390/healthcare10020258

**Published:** 2022-01-28

**Authors:** Mihaela Corlade-Andrei, Cornelia Măirean, Paul Nedelea, Gabriela Grigorași, Diana Cimpoeșu

**Affiliations:** 1 Faculty of Medicine, University of Medicine and Pharmacy “Gr. T. Popa”, 700115 Iasi, Romania; corladeandrei.mihaela@yahoo.com (M.C.-A.); gabriela.tiulica@yahoo.com (G.G.); dcimpoiesu@yahoo.com (D.C.); 2 Emergency “St. Spiridon” Hospital, 700111 Iasi, Romania; paul.nedelea@yahoo.com; 3 Faculty of Psychology and Educational Sciences, Alexandru Ioan Cuza University, 700554 Iasi, Romania

**Keywords:** burnout, healthcare professionals, COVID-19 pandemic, emergency staff

## Abstract

Objective: The aim of this current study was to identify the prevalence of burnout manifestation in a sample recruited from the emergency department of a hospital. Moreover, we aimed to assess the role of professional experience, age, and the professional category in shaping burnout manifestations. Results: The results showed that higher proportions of burnout symptoms were reported by resident physicians, nurses, and physicians whereas lower proportions were encountered in the orderly group. Moreover, the results revealed a significant difference between men and women in the nurse group concerning depersonalization; men presented higher levels of depersonalization compared with women. Concerning emotional exhaustion and depersonalization, multiple comparisons showed differences among the professional categories. Conclusions: The implications of these results for preventing burnout syndrome are discussed.

## 1. Introduction

In recent years, burnout syndrome has been a widely discussed issue among medical staff. At almost two years after the onset of the biggest pandemic of the modern world and one year after the first anti-COVID vaccine became available, the literature points to increasing burnout among healthcare workers all over the word [1,2,3]. Before the COVID-19 pandemic, the statistics showed that more than 400 doctors took their own lives every year but now, due to depression and exhaustion, there has been a sharp increase in these statistics [4]. This pandemic has raised global public health concerns and has required a reorganization of the healthcare system. Considering the context, chronic physical exhaustion and burnout symptoms have become increasingly pronounced. Medical professionals (e.g., physicians and nurses) from different specialties (emergency medicine, intensive care, infectious diseases, and pneumology) are even more exposed due to a higher workload and prolonged exposure to the risks of the virus [5,6].

Although emotional protective factors have been identified and proactive psychological strategies have been implemented to maintain the positive psychological tonus of the people working in healthcare, people around the world have been factually marked by the major changes imposed by the pandemic. When the coronavirus outbreak started, information on the transmissibility of the virus was limited and the focus was on informing the population about hygiene, social distancing, and measures taken by each country to prevent transmission within their borders. During this period, healthcare workers remained highly exposed to the virus due to prolonged contact with infected patients and conventional protection often proved to be insufficient. For example, a patient undergoing surgery in a hospital in Wuhan, China, was the source of infection for 14 healthcare workers even if the patient did not show symptoms suggestive of a SARS-CoV-2 infection [7].

According to a British survey conducted in 2019, approximately 80% of physicians were identified with a high risk of burnout [8]. Data from the literature highlight a two-fold risk of suicidal tendencies among physicians compared with the general population in the United States [9]. A recent study showed that Romanian medical residents presented an average burnout of 76% approximately two months after the pandemic outbreak in Romania [6]. A recent literature review documented the highest rates of burnout among nurses during the COVID-19 pandemic compared with the pre-pandemic period [3]. Moreover, in an empirical study that compared different medical professional categories (i.e., nursing, medical, allied health, and support staff represented by clerks, security, cleaners, and porters), nurses presented a higher level of emotional exhaustion [2]. Another study also showed that emotional exhaustion was the most frequently reported burnout dimension among nurses during the first wave of the COVID-19 pandemic [5]. Other comparisons between nurses and physicians sustain the fact that nurses are more likely than physicians to report burnout [10].

Previous literature has focused on the psychological risks of health care workers related to other epidemics in detecting high levels of anxiety, depression, stress, and burnout. Symptoms such as anxiety and fear increased immediately in the early stages of an epidemiological crisis but declined rapidly in the later stages whereas depression and post-traumatic stress symptoms persisted over time. Frontline healthcare workers involved in the diagnosis, treatment, and care of patients with COVID-19 reported more severe symptoms of anxiety, depression, and stress than those not on the frontline [1]. Burnout correlates with depression and anxiety, which can also make the struggle more difficult or worse [11].

Emergency medical care has its unique peculiarities and this uniqueness often leads to vicarious trauma manifestation and secondary traumatic stress [12,13]. It implies a high workload and exposure to a variety of stressors in an unpredictable way [2]. A survey conducted over four years and published in 1996 found that 60% of the respondents were in the moderate to high burnout ranges, more than 10% higher than internal medicine specialists and nearly 20% higher than the average rate of the respondents. Although burnout in the emergency department has continued, the latest Medscape report indicates that emergency medicine is at the top of the most stressful specialties. As in other specialties, burnout sets in early with studies highlighting that between 65–74% of residents (all levels) manifest symptoms of burnout [8]. In the context of the current pandemic, studies have also revealed high levels of burnout among emergency staff [2,14,15].

Based on these theoretical premises, and especially in the current context, we consider that it is important to assess the psychological status of medical staff working in an emergency department. This is one of the few studies conducted in Romania for this category of healthcare workers. Our main objective was to analyze the risk or the presence of burnout among the medical personnel who worked in an emergency department from the outbreak of the pandemic to the present. We also aimed to compare medical categories in terms of burnout dimensions. Based on previous literature presented above [2,10], we expected to find a higher rate of burnout among nurses compared with physicians, medical residents, and orderlies.

## 2. Materials and Methods

### 2.1. Participants and Procedure

The research took place within a university hospital in the city of Iasi, Romania, in the emergency department during the summer of 2021. A comparative design was used and all staff working in this department were invited to participate in this study. As a sampling strategy, we used self-selection sampling where the participants volunteered to take part in the research of their own accord. Permission to administer the survey was obtained from the institutional review board of the hospital and informed consent was obtained from all of the participants. They were informed that their participation was voluntary and then they were asked to complete a questionnaire battery on a confidential basis. The importance of answering truthfully was emphasized. Only the participants from the Emergency Unit were included in the sample. There were no exclusion criteria based on demographic variables. The study questionnaires were distributed in paper and pencil format and the participants were asked to return the completed questionnaires within one week. The participants completed all the measures anonymously. To encourage honest and valid responses and to prevent participant identification, the raw data were processed and analyzed by a researcher who was not affiliated with the medical unit. The participants were not remunerated but were told that they could be informed about the results of the study.

### 2.2. Instrument

In order to measure burnout, we used the Maslach Burnout Inventory [16], a widely used 25-item self-reporting instrument comprising three subscales: emotional exhaustion, depersonalization, and lack of personal accomplishment. The participants indicated how frequently they experienced each symptom in the present using a 5-point scale (1 = rarely; 5 = very often), providing scores for emotional exhaustion (9 items, e.g., “I feel emotionally drained from my work”), depersonalization (6 items, e.g., “I don’t really care what happens to my colleagues”) and lack of personal accomplishment (10 items, e.g., “I can easily create a relaxed atmosphere at my work”, reversed item). A total score could also be computed. Higher scores indicated a higher level of burnout. In order to verify the factorial validity of the scale, we applied a confirmatory factor analysis in AMOS Graphics 22 [17]. For the model fit, we applied the maximum likelihood estimation and reported the following fit indexes: the chi-squared statistic (χ2); the comparative fit index (CFI); and the root mean square error of approximation (RMSEA). A RMSEA < 0.05, χ^2^/df < 3, and CFI > 0.90 indicated a very good model fit [18]. The model with three factors fitted the data to a satisfactory degree: χ^2^(205) = 292.11, *p* < 0.001; CFI = 0.91; and RMSEA = 0.06, 95% CI (0.04–0.08). Thus, in this present study we computed three separate scores for each dimension of burnout. The Cronbach Alpha coefficients were 0.78 for emotional exhaustion, 0.82 for depersonalization, and 0.70 for lack of personal accomplishment.

The demographic information was collected via a questionnaire that covered age, gender, and occupation.

## 3. Results

### 3.1. Sample Characteristics

The participants in this study were made up of 97 medical and auxiliary workers consisting of 18.6% physicians, 14.4% resident physicians, 47.4% nurses, 7.2% orderlies, 10.3% stretcher-bearers, and 2.1% registrars. Most of the sample was composed of women (73.2%). The ages ranged from 24 to 53 with a mean age of 37.72 years; SD = 7.20. The demographic characteristics of the participants are presented in Table 1.

### 3.2. Preliminary Analysis

Based on a Kolmogorov–Smirnov Z test for normality [19], only the emotional exhaustion dimension of burnout was normally distributed. The other two dimensions, depersonalization and lack of personal accomplishment, were normalized in order to meet the assumptions for the parametrical analysis. After the normalization procedure, all the scores from the three dimensions of burnout (emotional exhaustion, depersonalization, and lack of personal accomplishment) were normally distributed (*p* > 0.05 for all variables).

Table 2 summarizes the self-reported burnout symptoms for the total sample as well as for each professional category. Only answers that indicated the occurrence of a symptom often and very often were considered. Generally, higher proportions of burnout symptoms were reported by resident physicians, nurses, and physicians whereas the lower proportions were reported in the orderly group. When analyzing the total sample, the most reported burnout indicators were “being at the power limit” (56.7%) and “feeling used up at the end of the workday” (30.9%). However, at the same time, a considerable proportion of the participants (72.2%) reported optimism concerning the achievement of future plans. For the total sample, the symptoms with the lower frequencies of occurrence were “threating others as objects” (2%) followed by “finding the right solution” (3.1%), “lack of interest” (5.1%), and “feeling overwhelmed by the situation” (5.2%). The percentages for each professional category can be seen in Table 2.

### 3.3. Differences between the Professional Categories concerning Burnout Manifestations

Overall, orderlies reported lower levels of several burnout manifestations compared with physicians, resident physicians, and nurses. Moreover, orderlies presented higher scores for “finding the right solution” and lower scores for “lack of interest” compared with stretcher-bearers. No significant differences were found among physicians, resident physicians, and nurses nor between stretcher-bearers and the medical professional categories (physicians, resident physicians, and nurses). These results are presented in Table 3.

### 3.4. Associations among the Study Variables

Pearson correlations were conducted in order to identify the associations between age and the three dimensions of burnout. The results showed that age was not significantly associated with any of the three dimensions (all *p* > 0.05). Emotional exhaustion was positively related to depersonalization (r = 0.76, *p* < 0.001) and lack of personal accomplishment (r = 0.43, *p* < 0.001). Further, there was a positive relation between personalization and lack of personal accomplishment (r = 0.34, *p* < 0.001). The relations were medium and strong [20]. Thus, the participants that reported high levels of emotional exhaustion also reported high levels of depersonalization and a lack of personal accomplishment. The results are presented in Table 4.

### 3.5. Gender Differences concerning Burnout Dimensions

An independent sample *t*-test analysis was conducted to examine the differences between women and men concerning emotional exhaustion, depersonalization, and lack of personal accomplishment. The results revealed that there were no statistically significant differences in the ratings of burnout dimensions when analyzing gender differences from the entire sample (all *p* > 0.05). Further *t*-test analyses were conducted to examine the gender differences separately for the professional categories of physicians, resident physicians, and nurses. An analysis was not possible for orderlies (the sample included only women), stretcher-bearers (the sample included only men), and registrars (the sample included only two participants). The results revealed a single significant difference between men and women in the nurse group concerning depersonalization; *t* (44) = 2.19, *p* = 0.038. Men presented higher levels of depersonalization (M = 1.15, SD = 0.09) compared with women (M = 1.05, SD = 0.18). All the other differences were non-significant.

### 3.6. Differences among the Professional Categories concerning Burnout Dimensions

**Figure 1:**
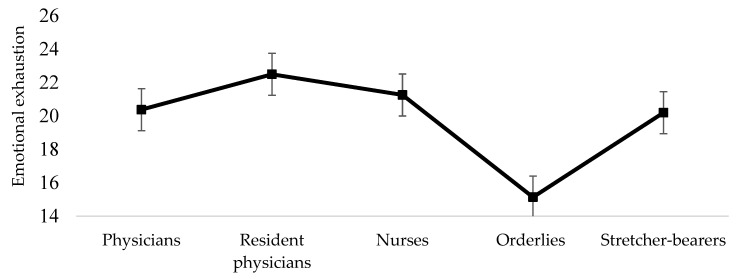
Differences among professional categories concerning emotional exhaustion.

In order to compare the different professional categories included in our sample concerning burnout dimensions, we used a one-way analysis of variance (ANOVA). Concerning emotional exhaustion, post-hoc multiple comparisons revealed significant differences between orderlies and the categories of physicians (M dif = −5.24, *p* = 0.046), resident physicians (M dif = −7.35, *p* = 0.010), and nurses (M dif = −6.12, *p* = 0.003). Orderlies (M = 15.14, SD = 2.79) presented a lower level of emotional exhaustion compared with physicians (M = 20.38, SD = 5.88), resident physicians (M = 22.50, SD = 6.08), and nurses (M = 21.26, SD = 6.12). Physicians did not significantly differ from resident physicians concerning emotional exhaustion (M dif = −2.11, *p* = 0.859). Nurses had similar levels of emotional exhaustion compared with physicians (M dif = 0.87, *p* = 0.984) and resident physicians (M dif = −1.23, *p* = 0.963). The results are presented in Figure 1.

**Figure 2:**
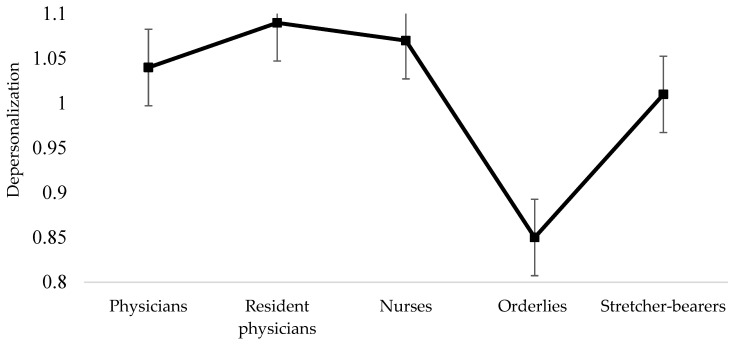
Differences among professional categories concerning depersonalization.

The results of the comparisons among the professional categories concerning depersonalization (Figure 2) also revealed significant differences between orderlies and resident physicians (M dif = −0.23, *p* = 0.022), as well as between orderlies and nurses (M dif = −0.21, *p* = 0.015). Orderlies (M = 0.85, SD = 0.13) presented a lower level of depersonalization compared with resident physicians (M = 1.09, SD = 0.18) and nurses (M = 1.07, SD = 0.17). Physicians did not significantly differ from resident physicians (M dif = −0.05, *p* = 0.893) and nurses (M dif = −0.03, *p* = 0.954) and resident physicians did not differ from nurses (M dif = 0.02, *p* = 0.994).

**Figure 3:**
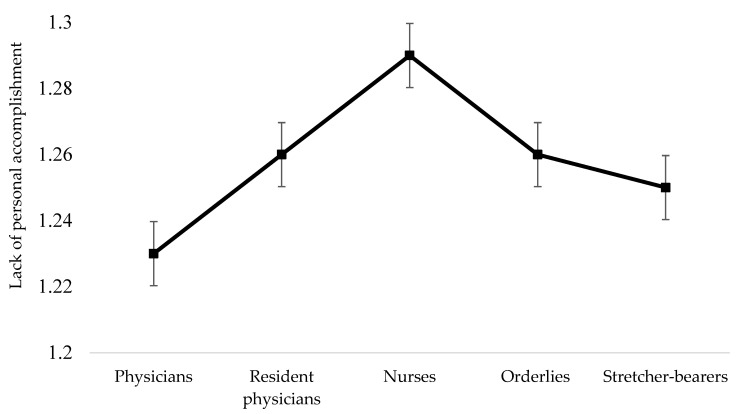
Differences among professional categories concerning personal accomplishment.

For personal accomplishment, the results showed that there were no significant differences among all the analyzed professional categories; F (4, 90) = 1.16, *p* = 0.330. These results are presented in Figure 3.

## 4. Discussion

This study aimed to examine the presence of burnout manifestations in a sample of healthcare providers from the emergency department of a hospital during the COVID-19 pandemic. The results showed that the most reported items were optimism concerning the achievement of future plans, being at the power limit, and feeling used up at the end of the workday. Despite a negative burnout manifestation, the participants also kept an optimistic perspective about the future. As other studies have suggested, professional quality of life includes not only negative indicators (e.g., burnout) but also the satisfaction derived from helping others in the present [21]. This satisfaction can generate self-confidence in personal abilities in order to fulfill the professional role in the future. Future studies addressing both the negative (i.e., secondary traumatic stress and compassion fatigue) and positive (i.e., compassion satisfaction) dimensions of professional quality of life are needed in order to understand the implications of challenging situations in the different professional categories.

Our results suggested that women and men were equally vulnerable for burnout, a similar result to other studies recently conducted with Romanian samples of medical staff during the COVID-19 pandemic [6,14]. When analyzing the differences among the different professional categories, we observed that orderlies presented lower levels of emotional exhaustion and depersonalization compared with the other professional categories (physicians, resident physicians, and nurses). However, among physicians, resident physicians, and nurses, the differences were not significant. Other studies have also reported no significant differences between physicians and nurses on indicators specific to professional quality of life (compassion satisfaction and secondary traumatic stress symptoms) [21]. Moreover, in our study, orderlies reported lower levels of several burnout symptoms (used up at the end of the day, overwhelmed, frustration, difficulties in finding the right solution, depression, lack of interest, feeling tense, work indifference, desire to isolate, created a benevolent atmosphere, and optimism for the future) compared with physicians, resident physicians, and nurses. Challenges imposed by the current pandemic might help us to explain these results. The responsibility of medical acts is higher and this can lead to a high overload for medical staff represented by physicians (including resident physicians) and nurses. For these reasons, particular events such as deaths of COVID-19 patients can be more difficult to manage by medical staff.

From a practical point of view, these results suggested a necessity to identify and implement personal and organizational strategies designed to prevent high rates of burnout during the COVID-19 pandemic and other detrimental consequences associated with this phenomenon. A few examples of interventions to prevent and manage intense distress that have been documented in previous studies as effective in dealing with stress and burnout are represented by mindfulness training, extending and using social support resources, and self-care (breaks, time with family and friends) [3]. Multifaceted programs designed to reduce stress and burnout as well as to increase wellbeing and resilience should also be implemented at an organizational level. Self-disclosure, psycho-education, the normalization of stress reactions, and validation are important elements to be targeted in these programs. Psychological interventions such as psychological debriefing after critical events (e.g., the death of a patient) can be an effective way to deal with intense emotional reactions generated by these events [22]. Beyond all the aspects that a pandemic entails, in the long term, this period should lead to a better understanding of the risk factors to which medical staff are exposed, particularly the medical teams in emergency departments.

When interpreting these results, several limitations should be considered. First, we used a limited sample of participants from only one hospital department. Thus, the current result cannot be generalized to other healthcare professionals from other hospital departments. Second, we did not explore other personal and professional factors (e.g., coping strategies, emotional regulation strategies) associated with burnout manifestation for a better understanding of the phenomenon. However, there are numerous studies about burnout syndrome that create a clear picture of burnout manifestations in different samples in relation to personal and social factors. This study only aimed to investigate the presence of the phenomenon in a highly exposed group in this very challenging period.

## 5. Conclusions

In conclusion, the results of this present study showed that participants reported a high frequency of optimism for the future and also specific burnout indicators such as power limit and feeling used up at the end of the day. Moreover, all the medical categories (physicians, resident physicians, and nurses) were affected despite the working experiences at a significantly higher level compared with non-medical staff (i.e., orderlies). Protecting healthcare workers should be one of the most important concerns of future prevention campaigns. Identifying risk factors and reporting them is extremely important for outlining future preventive strategies designed to reinforce the existing ones and, at the same time, to prevent possible crises in this domain.

## Figures and Tables

**Table 1:** Demographic and professional characteristics of the participants; *n* = 97.

Variables	*n*	%	M	SD
Age			37.72	7.90
Gender				
Female	71	73.2		
Male	26	26.8		
Profession				
Physicians	18	18.6		
Residential physicians	14	14.4		
Nurses	46	47.4		
Orderlies	7	7.2		
Stretcher-bearers	10	10.3		
Registrars	2	2.1		

**Table 2:** Proportion of the participants reporting the presence of burnout manifestations often and very often by professional categories.

Burnout Manifestations	Total Sample (%) *n* = 97	Physicians (%) *n* = 18	Resident Physicians (%) *n* = 14	Nurses (%) *n* = 46	Orderlies (%) *n* = 7	Stretcher-Bearers (%) *n* = 10
Emotionally drained	11.4	16.7	21.4	8.7	0	10
Used up	30.9	33.4	42.9	34.8	0	20
Fatigued in the morning	21.6	22.2	28.5	26	0	10
Overwhelmed	5.2	0	14.2	4.4	0	10
Impersonal ‘objects’	2	0	14.2	0	0	0
Frustrated	6.2	0	7.1	10.9	0	0
Full of energy *	17.5	11.2	21.4	19.5	14.3	20
Right solution *	3.1	0	0	4.3	14.3	0
Depression	6.2	0	14.3	8.7	0	0
Positive influence *	14.4	5.6	7.1	17.4	42.9	0
More callous	12.3	16.7	14.3	13.1	0	10
Lack of interest	5.1	5.6	7.1	4.3	0	10
Plans for the future *	9.3	0	0	15.2	0	10
Professional disillusions	7.2	5.6	0	13	0	0
Indifference	11.4	5.6	14.2	15.2	0	10
Tense	7.2	5.6	7.1	8.7	0	10
Work indifference	7.2	0	21.4	6.5	0	10
Want to isolate	15.5	11.2	21.4	21.8	0	0
Benevolent atmosphere *	9.3	0	7.1	15.2	0	10
Communicate easily *	6.2	0	7.1	4.4	14.3	20
Manage to do a lot *	6.2	0	7.1	4.4	14.3	20
Power limit	56.7	55.5	42.9	58.7	71.4	60
Optimism for the future *	72.2	77.8	92.9	67.4	42.9	70
Bankrupt	6.2	11.1	0	6.7	0	0
Burden on my shoulders	7.2	16.7	7.1	6.5	0	0

Note: * = reversed items.

**Table 3:** Means (and standard deviations) for burnout symptoms by professional categories.

Burnout Manifestations	Total Sample M (SD)	Physicians M (SD)	Resident Physicians M (SD)	Nurses M (SD)	Orderlies M (SD)	Stretcher-Bearers M (SD)
Emotionally drained	2.18	2.22	2.71	2.13	1.57	2.10
Used up	2.83	2.72	**3.21**	**3.00**	**1.42**	2.70
Fatigued in the morning	2.53	2.77	2.78	2.54	1.85	2.10
Overwhelmed	1.94	**2.11**	**2.57**	**1.89**	**1.14**	1.60
Impersonal ‘objects’	1.84	1.83	2.00	1.89	1.42	1.80
Frustrated	1.82	**1.61**	1.92	**2.00**	**1.00**	1.80
Full of energy *	2.49	2.27	2.64	2.65	2.14	2.20
Right solution *	2.26	**2.05**	**2.14**	**2.28**	**3.14**	**2.10**
Depression	1.65	1.27	1.92	**1.82**	**1.00**	1.80
Positive influence *	2.61	2.11	2.64	2.73	3.14	2.30
More callous	2.15	2.50	2.42	2.13	1.28	1.90
Lack of interest	1.89	1.61	**2.00**	**2.02**	**1.00**	**2.30**
Plans for the future *	2.05	1.66	1.64	2.32	1.71	2.10
Professional disillusions	1.89	1.83	1.85	2.06	1.28	1.70
Indifference	2.11	1.83	2.21	2.30	1.57	2.00
Tense	1.93	**1.94**	**2.14**	**1.93**	**1.14**	2.10
Work indifference	1.87	1.5	2.21	**2.08**	**1.00**	1.60
Want to isolate	2.27	2.22	**2.78**	**2.46**	**1.28**	1.60
Benevolent atmosphere *	2.34	2.27	2.28	**2.50**	**1.71**	2.20
Communicate easily *	2.01	1.77	1.92	2.04	2.00	2.30
Manage to do a lot *	2.11	1.66	2.21	1.27	2.57	2.30
Power limit	3.79	3.38	3.35	3.93	4.42	4.00
Optimism for the future *	3.71	3.71	**4.35**	**3.73**	**1.17**	3.60
Bankrupt	1.53	1.72	1.50	1.52	1.00	1.60
Burden on my shoulders	1.95	2.33	1.85	1.89	1.57	2.00

Note: Significant differences are in bold. * = reversed items.

**Table 4:** The associations among the variables.

Burnout Dimensions	1	2	3	4
1. Emotional exhaustion	1			
2. Depersonalization	0.76 *	1		
3. Personal accomplishment	0.43 *	0.34 *	1	
4. Age	−0.09	−0.09	−0.10	1

Note: * = *p* < 0.001.

## Data Availability

The raw data supporting the conclusions of this article are made freely available by the authors at the following address: 10.4121/17159390.
